# Long Non-Coding RNA in Cancer

**DOI:** 10.3390/ijms14034655

**Published:** 2013-02-26

**Authors:** Nina Hauptman, Damjan Glavač

**Affiliations:** Department of Molecular Genetics, Institute of Pathology, University of Ljubljana, SI-1000 Ljubljana, Slovenia; E-Mail: nina.hauptman@mf.uni-lj.si

**Keywords:** long non-coding RNA, cancer, oncogenic lncRNA, tumor suppressor lncRNA

## Abstract

Long non-coding RNAs (lncRNAs) are pervasively transcribed in the genome and are emerging as new players in tumorigenesis due to their various functions in transcriptional, posttranscriptional and epigenetic mechanisms of gene regulation. LncRNAs are deregulated in a number of cancers, demonstrating both oncogenic and tumor suppressive roles, thus suggesting their aberrant expression may be a substantial contributor in cancer development. In this review, we will summarize their emerging role in human cancer and discuss their perspectives in diagnostics as potential biomarkers.

## 1. Introduction

The central dogma of molecular biology postulates gene-coding through storage of genetic information and proteins as the main molecules of cellular functions, while RNA has the role of an intermediary between DNA sequence and encoded protein. The findings of the human genome project thus came as a surprise, since only 1.5% of the human genome encodes protein-coding genes [1–5]. Development of new techniques revolutionized the molecular world with evidence that at least 90% of the human genome is actively transcribed [6,7]. The human transcriptome has shown more complexity than previously assumed since the protein-coding transcripts are being a minority, compared to a more complex group of non-coding RNAs (ncRNAs), such as microRNAs (miRNAs), long non-coding RNAs (lncRNAs), small nucleolar RNAs (snoRNAs), small interfering RNAS (siRNAs), small nuclear (snRNAs), and piwi-interacting RNAs (piRNAs) [8–15]. Although initially thought to be transcriptional noise, ncRNA may play a crucial role in cellular development, physiology and pathologies.

**Figure 1:**
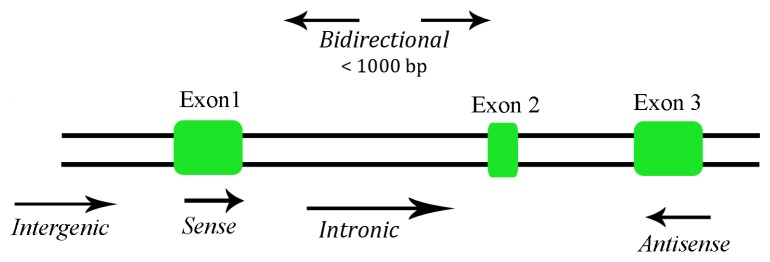
Categories of long non-coding RNA (lncRNA).

Depending on their size, ncRNAs are divided into two major groups. Transcripts shorter than 200 nucleotides are referred to as small ncRNAs, which include miRNA, siRNA, piRNA, *etc.* The other group is composed of lncRNA, where the transcripts lack a significant open reading frame, and have length of 200 nt up to 100 kilobases. A lncRNA can be placed into one or more of five broad categories: (1) sense, or (2) antisense, when overlapping one or more exons of another transcript on the same, or opposite, strand, respectively; (3) bidirectional, when the sequence is located on the opposite strand from a neighboring coding transcript whose transcription is initiated less than 1000 base pairs away, (4) intronic, when it is derived wholly from within an intron of a second transcript, or (5) intergenic, when it lies within the genomic interval between two genes [16] (Figure 1). There are some lncRNAs that are transcribed by RNA polymerase III while the majority of lncRNAs are transcribed by RNA polymerase II, spliced and polyadenylated [17]. Most of the lncRNAs are located in the cytoplasm, although there are some found in both cytoplasm and nucleus [18].

## 2. Long Non-Coding RNA Functions

LncRNAs are involved in almost every step of a life cycle of genes and regulate diverse functions. Several lncRNAs can regulate gene expression at various levels, including chromatin modification, transcription, and posttranscriptional processing [19].

So far, their role was extensively studied in epigenetic regulation, such as imprinting. Diploid organisms carry two alleles of each of the parents’ autosomal genes. In most cases, both of the alleles are expressed equally, except when a subset of genes shows imprinting in which expression is restricted by epigenetic mechanism to either maternal or paternal allele [17]. X-inactivation (XCI) is a process that equalizes gene expression between males and females by inactivating one X in female cells [17]. Some lncRNAs participate in global cellular behavior by controlling apoptosis, cell death and cell growth [15,20]. LncRNA can also mediate epigenetic modification by recruiting chromatin remodeling complex to specific chromatin loci, e.g., HOTAIR by polycomb repression complex 2 (PCR2) and/or lysine-specific demethylase 1 (LSD1), CCND1 by protein termed translocated in liposarcoma (TLS), and ANRIL by polycomb repression complex 1 and 2 (PCR1 and PCR2) [5,21–25]. The mode of action of some lncRNAs is interaction with their intracellular steroid receptors. Other lncRNAs function by regulating transcription through a variety of mechanisms that include interacting with RNA-binding proteins, acting as a coactivator of transcription factors, or repressing a major promoter of their target gene [22]. In addition to chromatin modification and transcriptional regulation, lncRNAs can regulate gene expression at the posttranscriptional level.

## 3. Oncogenic lncRNA

**SRA—Steroid Receptor RNA Activator** is a coactivator for steroid receptors and acts as an ncRNA found in the nucleus and cytoplasm. SRA regulates gene expression mediated by steroid receptors through complexing with proteins also containing steroid receptor coactivator 1 (SRC-1) [26]. The SRA1 gene can also encode a protein that acts as a coactivator and corepressor [27]. SRA levels have been found to be upregulated in breast tumors where it is assumed that increased SRA levels change the steroid receptors’ actions, contributing to breast tumorigenesis. While the expression of SRA in normal tissues is low, it is highly up-regulated in various tumors of the human breast, uterus and ovary. This evidence supports that SRA is a potential biomarker of steroid-dependent tumors [26].

**HOTAIR—HOX Antisense Intergenic RNA** with a length of 2.2 kb was found in the *HOXC* locus and is transcribed in antisense manner [28]. It is the first lncRNA discovered to be involved in tumorigenesis. In breast cancer, both primary and metastatic, the expression is up regulated; in the latter case up to 2000-fold increase was shown [23]. The high expression level of HOTAIR in primary breast cancer is also correlated to metastasis, and poor survival rate [23]. The level of HOTAIR expression is higher in patients with lymph node metastasis in hepatocellular cancer [29].

Polycomb group proteins mediate repression of transcription of thousands of genes that control differentiation pathways during development, and have roles in stem cell pluripotency and human cancer [23,30–34]. The target of PRC2 is the *HOXD* locus on chromosome 2 where the PRC2 in association with HOTAIR causes the transcriptional silencing of several metastasis suppressor genes resulting in breast epithelial cells having the expression of embryonic fibroblast. Alternating the level of HOTAIR results in enhanced PRC2 repressive activity [23]. HOTAIR acts as a molecular scaffold having two known chromatin modification complexes. The 5′ region of lncRNA binds to the PRC2 complex responsible for H3K27 methylation and the 3′ region binds to LSD1, which mediates enzymatic demethylation of H3K4 [24,30,35]. This result suggests the possible function of HOTAIR as a scaffold binding to selected histone modification enzymes and therefore causing histone modification on target genes [30]. Although the precise mechanism is still not known, it is clear that HOTAIR remodels chromatin to promote cancer invasiveness.

HOTAIR as an epigenetic regulator in gene expression is deregulated in different cancers [23,36–38]. In hepatocellular carcinoma (HCC) and HCC patients with liver transplantation, the levels of HOTAIR compared with normal liver tissue are elevated. Expression levels of HOTAIR can also be used as an independent prognostic marker for HCC recurrence and lower survival rate [31]. HOTAIR can be a potential biomarker for the existence of lymph node metastasis in HCC [29].

### ANRIL—Antisense ncRNA in the INK4 locus

Many transcripts coding for proteins have anti-sense partners, whose perturbation can alter the expression of the sense transcripts [39]. Some of these genes are tumor suppressors, which can be epigenetically silenced by antisense ncRNA [40].

ANRIL activates two polycomb repressor complexes, PRC1 and PRC2 [21,25], resulting in chromatin reorganization which silences the INK4b-ARF-INK4a locus encoding tumor suppressors p15^INK4b^, p14^ARF^, p16^INK4a^, which are active in cell cycle inhibition, senescence and stress-induced apoptosis. Overexpression of ANRIL in prostate cancer has shown silencing of INK4b-ARF-INK4a and p15/CDKN2B by heterochromatin reformation [25,41]. The repression is mediated by direct binding to combox 7 (CBX 7) and SUZ12, members of PRC1 and PRC2, respectively [21,25].

### MALAT 1—Metastasis-Associated Lung Adenocarcinoma Transcript 1

This lncRNA was first associated with high metastatic potential and poor patient prognosis during a comparative screen of non-small cell lung cancer patients with or without metastatic tumors [42]. MALAT1 is widely expressed in normal human tissues [42,43] and is found to be upregulated in a variety of human cancers of the breast, prostate, colon, liver and uterus [44–47]. The MALAT1 locus at 11q13.1 has been identified to harbor chromosomal translocation break points associated with cancer [48–50]. MALAT1 is localized in nuclear speckles and widely expressed in normal tissues [42,43], but was found to be upregulated in hepatocellular carcinoma, breast, pancreas, osteosarcoma, colon and prostate cancers [44–47,51]. It has been shown that increased expression of MALAT1 can be used as a prognostic marker for HCC patients following liver transplantation [52].

A number of studies have implicated MALAT1 in the regulation of cell mobility, due to its high levels of expression in cancers. For example, RNA interference-mediated silencing of MALAT1 reduced the *in vitro* migration of lung adenocarcinoma cells by influencing the expression of motility-related genes [53]. Recent studies on knockout MALAT1 mice have not displayed any cellular phenotype. Future studies will be needed where mice will be exposed to different stresses, such as induction of cancer, which will potentially unveil its function. It is known that MALAT1 as well as HOTAIR play vital roles in human cells but it is possible that they have no significant role in living animals under normal physiological conditions [54–56].

## 4. Oncogenic and Tumor Suppressor lncRNA

***H19*** is expressed from the maternal allele and has a pivotal role in genomic imprinting during cell growth and development [57]. The locus contains *H19* and insulin-like growing factor 2 (*IGF2*), which are imprinted. This leads to differential expression of both genes, *H19* from maternal and *IGF2* from paternal allele [57,58]. The loss of imprinting results in misexpression of *H19* and was observed in many tumors including hepatocellular and bladder cancer [59,60]. This lncRNA has been linked to oncogenic and tumor suppressor properties [57]. cMYC induces the expression of *H19* in different cell types where *H19* potentiates tumorigenesis [58]. In addition c-MYC also down-regulates expression of *IGF2* imprinted gene. *H19* transcripts are precursors for miR-675 which functionally down-regulates the tumor suppressor gene for retinoblastoma in human colorectal cancer [61]. Data support *H19* deregulation causing either oncogenic or tumor suppressor properties, although the exact mechanism is still elusive.

## 5. Tumor Suppressor lncRNA

### MEG3—Maternally Expressed Gene 3

LncRNA MEG3 is a transcript of the maternally imprinted gene. In normal pituitary cells MEG3 is expressed, the loss of expression is observed in pituitary adenomas and the majority of meningiomas and meningioma cell lines [62,63]. MEG3 activates regulation of tumor suppressor protein p53. Normally, p53 protein levels are extremely low due to its rapid degradation via the ubiquitin-proteasome pathway. The ubiquitination of p53 is mainly mediated by MDM2, an E3 ubiquitin ligase. MEG3 down-regulates MDM2 expression, which suggests that MDM2 down-regulation is one of the mechanisms whereby MEG3 activates p53 [64]. MEG3 significantly increases p53 protein level and stimulates p53-dependent transcription [65]. MEG3 enhances p53 binding to target promoters such as GDF15 but not p21 and is also able to inhibit cell proliferation in the absence p53, suggesting that MEG3 is a p53 dependent and independent tumor suppressor [62–65].

**GAS5—Growth Arrest-Specific 5** is widely expressed in embryonic and adult tissues. Expression is almost undetectable in growing leukemia cells and abundant in saturation density-arrested cells [66,67]. GAS5 functions as a starvation or growth arrest-linked riborepressor for the glucocorticoid receptors by binding to their DNA binding domain inhibiting the association of these receptors with their DNA recognition sequence. This suppresses the induction of several responsive genes including the gene encoding cellular inhibitor of apoptosis 2 (cIAP2), reducing cell metabolism and synthesizes cells to apoptosis [4,67]. GAS5 can induce apoptosis directly or indirectly in the prostate and breast cancer cell lines, where it was shown that GAS5 has a significantly lower expression in breast cancers compared to normal breast epithelial tissues [68].

**CCND1/Cyclin D1** is a heterogenous lncRNA transcribed from the promoter region of the Cyclin D1 gene. Cyclin D1 is a cell cycle regulator that is frequently mutated, amplified and over expressed in a variety of cancers [69]. LncRNA recruits the RNA-binding proteinTLS, which is a key transcriptional regulatory sensor of DNA damage signals. Upon binding TLS undergoes allosteric modification, modulating activities of CREB-binding protein (CBP) and p300, resulting in inhibition of the cyclin D1 gene expression [22].

**LincRNA-p21** expression is directly induced by the p53 signaling pathway. It is required for global repression of genes that interfere with p53 function to regulate cellular apoptosis. Lincrna-p21 mediated gene repression occurs through physical interaction with RNA-binding protein hnRNP K leading to the promoters of genes being repressed in a p53 dependent manner [70].

## 6. Diagnostic Benefits of lncRNA

So far, the majority of cancer biomarkers are protein-coding genes, their transcripts or the proteins. The non-coding regions are evolving as a biomarker hotspots only recently. By the advent of high-throughput sequencing, we are now able to identify deregulated expression of transcriptome at much higher resolution, what allow us to decipher smaller changes in the expression level. In the case of lncRNAs, where their main function is regulation of other genes expression, the importance of lncRNAs maintained expression is evident. Since cancer is a complicated disease, which involves many factors, molecular biomarkers are valuable diagnostic and prognostic tools that could ease the disease management. Compared to protein-coding RNAs, using lncRNA as markers is of advantage since their own expression is a better indicator of the tumor status. Many lncRNAs are now connected to cancer due to new technologies and are emerging into the field of molecular biology as new regulatory players. Several lncRNA were found to be deregulated in a wide variety of cancers (Table 1).

In breast cancer research higher expressions of SRA and SRAP, compared to normal tissue were observed. Possibly SRAP expression contributes to higher survival for patient undergoing Tamoxifen treatment [90].

The expression of MALAT 1 is elevated in osteosarcoma patients with poor response to chemotherapy, which suggests that this transcript plays a crucial role in the pathology of tumors [53].

Additionally MALAT 1 serves as an independent prognostic marker for patient survival in early stage non-small cell lung cancer [42].

In hepatocellular carcinoma, (HCC) definitive diagnosis of lymph node metastasis is difficult without histological evidence. It has been demonstrated that a significant correlation between *HOTAIR* gene expression and lymph node metastasis exists, suggesting that measuring HOTAIR lncRNA is a potential biomarker for predicting lymph node metastasis [29]. Upregulation of HOTAIR is closely associated with gastrointestinal stromal tumor (GIST) aggressiveness and metastasis and it can be used as a potential biomarker [38,91].

MALAT1 is a powerful biomarker for HCC recurrence prediction following liver transplantation. Moreover, silencing MALAT1 activity in HCC would be a potential anticancer therapy to prevent tumor recurrence after orthotopic liver transplantation [52].

SPRY4-IT1 expression is substantially increased in patient melanoma cell samples compared to melanocytes. The elevated expression of SPRY4-IT1 in melanoma cells, its accumulation in the cell cytoplasm, and its effects on cell dynamics suggest that the misexpression of SPRY4-IT1 may have an important role in melanoma development, and could be an early biomarker and a key regulator for melanoma pathogenesis in humans [85].

The novel potential biomarkers can be discovered through certain types of highly expressed cancer-associated lncRNAs [92]. Therapeutic benefit can be obtained through pathways mediating transcriptional gene silencing, especially those of tumor suppressors and oncogenes [93]. For patients’ comfort, biomarkers should be detected in samples obtained in a non-invasive way. Desirable samples are body fluids, such as serum or urine, where circulating nuclear acids (CNAs), both DNA and RNA species, are found. CNAs are found in plasma, cell-free serum, sputum and urine [29,94–97].

PRNCR1 (prostate cancer non-coding RNA1) expression was upregulated in some of the prostate cancer cells as well as precursor lesion prostatic intraepithelial neoplasia and considered as a tumor marker [75].

Suggestions that lncRNA can be used as biomarkers and/or drug targets have arisen from numerous studies observing the expression patterns of tumor tissues comparing to normal ones [14]. The possible therapies arising from this knowledge would be beneficial in cases where protein target drugs have not been effective. A recent study has shown that reduced expression of ncRAN enhanced the chemotherapeutic drug *in vitro*[98]. This opens another possibility of cancer treatment, where a combination of drugs would have much higher effect.

Often lncRNAs exhibit tissue specific patterns that distinguish them from miRNAs and protein-coding mRNAs that are expressed from multiple tissue types. Their specificity makes them precise biomarkers for cancer diagnostics [99]. PCA3 is a prostate-specific lncRNA overexpressed in prostate cancer. Although its functions are not understood, it was still utilized as a biomarker in a clinical test. Expression of the PCA3 transcript is determined from prostate cells in urine samples of patients [100,101]. Another lncRNA detected in body fluids is HULC, expression of which is disrupted in hepatocellular carcinomas and can be monitored in patients’ blood sera [102].

To understand the biology of cancer it will be essential to identify, annotate lncRNAs and study their expression profiles in human tissues and diseases [103,104]. With this, the potential of lncRNAs on biology and medicine will be revealed. Long non-coding RNAs have recently arisen as new discoveries in the field of molecular biology. Since only a few individual lncRNAs have been functionally studied, still a lot of questions remain to be addressed [4]. At the moment the full potential of cancer therapy is not yet developed. The future of it lies in specific targeting of cancer cells and specific delivery of the drugs. LncRNAs are a possible resource for developing diagnostics and therapies, although a better understanding of their function and precise mechanism through which they function are needed first [4]. Another possibility for cancer treatment lies in combination of drugs, where one would change the expression of lncRNA in a way for chemotherapeutic drug to have a higher effect. Since probably the lncRNA function through their secondary structure special molecules could be developed to disrupt their secondary structure or bind to them to form complexes through which an inactivation of lncRNA would occur. These molecules should be highly specific in order not to disrupt other molecules and mechanisms. To discover the right molecules more studies of the complex mechanisms involving lncRNA are needed.

## 7. Conclusions

RNA used to be just a messenger between coding genes and proteins encoded by them. However, “transcriptional noise” is turning out to be a very important part of regulation processes. With the discovery of LncRNA and their functions, the new world of molecular biology is emerging. There is much research still on the way towards a deeper understanding of regulation processes in which lncRNA is one of the important players. LncRNA deregulation in human disease is unveiling the complexity of cellular processes. Studying the mechanisms of lncRNA involvement in oncogenic and tumor suppressive pathways will lead to new cancer diagnostic markers and will pave the way to novel therapeutic targets.

## Figures and Tables

**Table 1:** Cancer associated lncRNAs (adapted from [15,71]).

Name	Cytoband (Size)	Cancer Types	References
AK023948	8q24.22 (2807 nt)	Papillary thyroid carcinoma (down regulated)	[72]
ANRIL	9p21.3 (~3.9kb)	Prostate, leukemia	[36,41]
BC200	2p21 (200 nt)	Breast, cervix, esophagus, lung, ovary, parotid, tongue	[73,74]
PRNCR1	8q24.2 (13 kb)	Prostate	[75]
*H19*	11p15.5 (2.3 kb)	Bladder, lung, liver, breast, esophagus, choriocarcinoma, colon	[57,58,76–80]
HOTAIR	12q13.13 (2.2 kb)	Breast, hepatocellular	[23,29,30,36]
HULC	6p24.3 (~500 nt)	Hepatocellular	[4,81,82]
LincRNA-p21	~3.1 kb	Represses p53 pathway; induces apoptosis	[70]
Loc285194	3q13.31 (2105 nt)	Osteosarcoma	[83]
Malat1	11q13.1 (7.5 kb)	breast, prostate, colon, liver, uterus	[44–47]
MEG3	14q32.2 (1.6 kb)	Brain (down-regulated)	[62,65]
PTNEP1	9p13.3 (3.9 kb)	Prostate	[84]
Spry4-it1	5q31.3 (~700 nt)	Melanoma	[85]
SRA	5q31.3 (1965 nt)	Breast, uterus, ovary (down-regulated)	[26,27]
UCA1/CUDR	19p13.12 (1.4, 2.2, 2.7 kb)	Bladder, colon, cervix, lung, thyroid, liver, breast, esophagus, stomach	[86,87]
Wt1-as	11p13 (isoforms)	acute myeloid leukemia	[88]
PCA3	9q21.22 (0.6–4 kb)	Prostate	[89]
GAS5	1q25.1 (isoforms)	Breast (down-regulated)	[68]
